# Recombinant monoclonal antibodies for labeling tubulin post-translational modifications in *Caenorhabditis elegans*

**DOI:** 10.17912/micropub.biology.002031

**Published:** 2026-03-25

**Authors:** Leah Dobossy, Juan Wang, Kristen J. Verhey, Maureen Barr

**Affiliations:** 1 Genetics, Rutgers University, New Brunswick, NJ, USA; 2 Cell Biology, University of Michigan School of Medicine, USA

## Abstract

Post-translational modifications (PTMs) of tubulin regulate microtubule properties and functions. The Verhey lab created recombinant monoclonal antibodies (rMAbs) against tubulin acetylation (rMAb-6-11B-1), tyrosinated α-tubulin (rMAb-YL1/2), and glutamylation (rMAb-GT335) (Blasius et al., 2025; Hotta et al., 2026). Here, we validate these rMAbs in *C. elegans* hermaphrodites and males. These rMAbs faithfully reproduce the reported cell-type-specific staining patterns of commercial antibodies, providing high-quality, cost-effective resources for studying the tubulin code.

**Figure 1. Staining for Tubulin post-translational modifications in the C. elegans nervous system:**
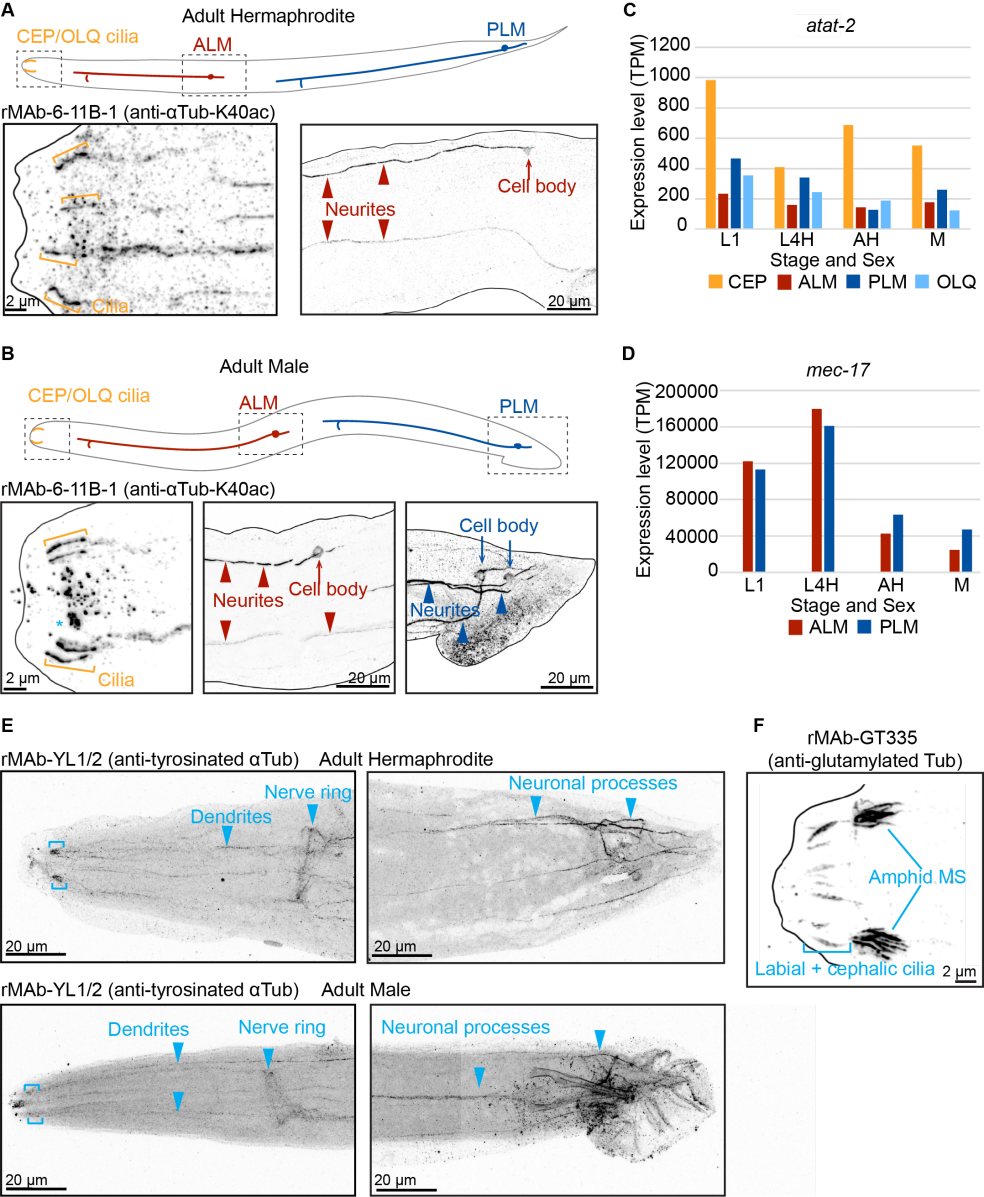
**(A-B)** Representative immunostaining of *C. elegans* hermaphrodites and males with rMAb-6-11B-1 (anti-αTub-K40ac). Cartoons show neuronal positions; rectangles indicate imaged regions. Strong staining is observed in curved cilia corresponding to CEP and OLQ cilia (orange brackets) in both hermaphrodites (A) and males (B). Scale bars=2μm. Asterisk indicates suspected background staining in the buccal cavity. Neuronal cell body (arrows) and processes (arrowheads) corresponding to ALM (red) and PLM (blue) mechanosensory neurons are detected in both hermaphrodites (A) and males (B). Scale bars=20μm. **(C-D)** α-Tubulin acetyltransferase genes *atat-2* (C) and *mec-17* (D) mRNA are detected at the highest levels in CEP and touch neurons. **(C)** *atat-2* is most highly expressed in CEP neurons across life stages and in both hermaphrodites and males. *atat-2* is also expressed in touch neurons and OLQ neurons at lower but potentially functional levels. **(D)** *mec-17* shows the highest expression in ALM and PLM neurons across developmental stages: larval stage 1 (L1), larval stage 4 hermaphrodite (L4H), adult hermaphrodite (AH), and adult male (M). *mec-17* is also expressed in CEP and OLQ at negligible levels. Expression values in touch neurons reach ~100,000 transcripts per million (TPM), while CEP and OLQ expression remains in the tens of TPM. Data obtained from the CeNGEN project (see Methods). **(E)** Representative immunostaining of anti-tyrosinated α-tubulin (rMAb-YL1/2) in *C. elegans* hermaphrodites (top) and males (bottom). Tyrosinated tubulin was mainly detected in dendrites and neuronal processes (arrowheads) in head and tail ganglia of both sexes. In the nose, tyrosinated tubulin is detected in regions that may be amphid cilia (bracket). Scale bars=20 μm. **(F)** Representative immunostaining of anti-glutamylated tubulin (rMAb-GT335) in *C. elegans* hermaphrodites. Glutamylated tubulin is detected in labial and cephalic cilia (bracket) and the middle segments (MS) of amphid channel cilia. Scale bar=2μm. All images represent maximum intensity z-projections.

## Description

Tubulin subunits of microtubules are decorated with a diverse array of post-translational modifications (PTMs) in a cell-specific manner. These tubulin PTMs constitute a "Tubulin Code" that dictates both intrinsic properties of microtubule structure, dynamics, and function and extrinsic interactions with microtubule-associated proteins (MAPs) (Janke & Magiera, 2020; Verhey & Gaertig, 2007). Monoclonal antibodies 6-11B-1 (anti-α-tubulin K40 acetylation), YL1/2 (anti-α-tubulin tyrosination) and GT335 (anti-tubulin glutamylation) have been invaluable research resources. Recently, recombinant monoclonal antibody (rMAb) versions of these antibodies have been developed and offer reduced cost, high quality, and increased reproducibility (Blasius et al., 2025; Hotta et al., 2026). However, the specificity of these rMAbs in *C. elegans* remained uncharacterized. Through immunofluorescence staining of *C. elegans* in both sexes, we demonstrate that rMAb-6-11B-1 specifically labels four curved cilia (likely CEP and/or OLQ) in the nose and the neuronal cell body and processes of touch neurons in the body, rMAb-YL1/2 labels neuronal processes in the head and tail, and rMAb-GT335 labels the middle segment of amphid cilia as well as labial and cephalic cilia, matching the specificity and activity of commercially available antibodies (Akella et al., 2010; Barbosa et al., 2017; Bayansan et al., 2025; Power et al., 2020; Shida et al., 2010). Our work provides the *C. elegans* community with high-specificity, high-affinity PTM labeling methods for tubulin acetylation, tyrosination, and glutamylation.

The 6-11B-1 antibody recognizes acetylation of the K40 residue of α-tubulin. In adult hermaphrodites, rMAb-6-11B-1 labels four curved cilia, likely CEP and/or OLQ cilia in the head, as well as ALM and PLM cell bodies and neurites ( Fig. 1A ), confirming previous findings using the commercial 6-11B-1 antibody (Akella et al., 2010; Shida et al., 2010). A similar staining pattern is observed in adult males ( Fig. 1B ).

Consistent with the staining patterns we observed, CeNGEN (Hammarlund et al., 2018), single-cell RNA-seq data show that the two α-tubulin acetyltransferase genes, *atat-2* and *mec-17* , are expressed at the highest levels in CEP and touch neurons, respectively. *atat-2* is most highly expressed in CEP neurons and at lower levels in OLQ neurons as well as the touch neurons ALM and PLM ( Fig. 1C ). Since OLQ and CEP cilia are closely located in the head and share similar morphology, we label the four cilia as CEP and/or OLQ in the head ( Fig. 1A- B). Whether OLQ ciliary microtubules are acetylated requires further cell-type-specific investigation.

In contrast, *mec-17* shows negligible expression in CEP and OLQ neurons (~tens of transcripts per million (TPM)) but is highly expressed in touch receptor neurons (~100,000 TPM) throughout all life stages and in both sexes ( Fig. 1D ). These expression patterns suggest that ATAT-2 is the primary α-tubulin acetyltransferase in CEP and/or OLQ neurons, whereas MEC-17 predominates in touch receptor neurons, consistent with previous findings that both enzymes are required for K40 α-tubulin acetylation and touch response in *C. elegans* (Akella et al., 2010; Shida et al., 2010).

The YL1/2 antibody recognizes the terminal tyrosine on the C-terminal tail of most α-tubulin isotypes. Previous studies using the commercial YL1/2 antibody in *C. elegans* showed that tyrosinated microtubules occur in the mitotic spindle of the one-cell embryo and to a lesser extent in the touch receptor neuron cell bodies (Barbosa et al., 2017; Lu et al., 2024). A separate study using a different clone of the anti-tyrosinated tubulin MAb (clone TUB-1A2) showed that tyrosinated microtubules also occur in the nerve ring and body neurons, co-localizing with UNC-104 puncta (Bayansan et al., 2025). We find that in both adult hermaphrodites and males, rMAb-YL1/2 labels dendrites and neuronal processes in the nerve ring and tail ganglia ( Fig. 1E ), largely consistent with previous findings (Bayansan et al., 2025). In addition, we observed neuronal dendrites exhibiting bilateral symmetry with a bundled morphology in the nose region, suggesting labeling of the base and/or middle segment (MS) of amphid channel cilia (Fig.1E, arrow). Co-localization with additional markers is required to confirm the identity of these rMAb-YL1/2-positive neurons and cilia.

The GT335 antibody recognizes branch-point glutamylation and has been extensively characterized in *C. elegans* cilia (Kimura et al., 2010, 2018; O'Hagan et al., 2011, 2017; Pathak et al., 2007; Power et al., 2020, 2024; Wolff et al., 1992). As expected, rMAb-GT335 labels the doublet region/middle segment (MS) of amphid channel cilia and the labial and cephalic cilia in the adult hermaphrodite head ( Fig. 1F ).

In summary, we have validated recombinant monoclonal antibody versions of three widely used tubulin PTM antibodies—rMAb-6-11B-1 (anti-acetylated α-tubulin), rMAb-YL1/2 (anti-tyrosinated α-tubulin), and rMAb-GT335 (anti-glutamylated tubulin)—in *C. elegans* . Our immunofluorescence analysis demonstrates that these rMAbs faithfully reproduce the staining patterns of their commercial counterparts, labeling distinct neuronal populations and subcellular compartments in both hermaphrodites and males. These high-quality and cost-effective rMAbs represent valuable tools for the *C. elegans* research community to investigate the spatial and temporal dynamics of tubulin post-translational modifications in neuronal development, function, and disease modeling.

## Methods

*Worm preparation and permeation*

The immunostaining protocol was adapted from Finney & Ruvkun (1990). Briefly, synchronized one-day-old adult worms were collected into 1.5 mL microcentrifuge tubes and washed several times with M9 buffer over 1 h on ice until the supernatant was clear to remove bacteria. The worm pellet was resuspended in 500 µL M9 buffer, and 500 µL of 2× ice-cold Ruvkun buffer (160 mM KCl, 40 mM NaCl, 20 mM EGTA, 10 mM spermidine-HCl, 30 mM PIPES, pH 7.4, and 50% methanol) supplemented with 4% formaldehyde was added. To crack the cuticle, tubes were rapidly frozen in liquid nitrogen and thawed under tap water. Worms were then washed twice with Tris-Triton buffer (100 mM Tris-HCl, pH 7.4, 1% Triton X-100, and 1 mM EDTA), resuspended in Tris-Triton buffer containing 1% β-mercaptoethanol, and incubated overnight at 37°C.

The following day, worms were washed with 1× BO₃ buffer (50 mM H₃BO₃, 25 mM NaOH) containing 0.01% Triton X-100 and incubated in 1× BO₃ + 0.01% Triton X-100 buffer supplemented with 10 mM DTT for 15 min at room temperature with gentle agitation. Worms were then washed and incubated in 1× BO₃ + 0.01% Triton X-100 buffer containing 0.3% H₂O₂ for 15 min at room temperature with gentle agitation. After an additional wash with 1× BO₃ + 0.01% Triton X-100 buffer, worms were blocked for 1 h in Antibody Buffer A (1× PBS, 1% BSA, 0.5% Triton X-100, 0.05% sodium azide, and 1 mM EDTA) at 4°C with gentle agitation.

*Antibody staining*

Animals were incubated with primary antibodies (rMAb-6-11B-1, rMAb-YL1/2, or rMAb-GT335) diluted 1:1000 in Antibody Buffer A for 24 h at 4°C with gentle agitation. Worms were washed with several changes of Antibody Buffer B (1× PBS, 0.1% BSA, 0.5% Triton X-100, 0.05% sodium azide, and 1 mM EDTA) over two hours at room temperature with gentle agitation. After a brief rinse with Antibody Buffer A, worms were incubated with Alexa Fluor 568-conjugated donkey anti- mouse secondary antibody (Cat. A10037; Invitrogen) diluted 1:1350 in Antibody Buffer A overnight at 4°C with gentle agitation. Worms were then washed with 5-6 changes of Antibody Buffer B over two hours before being mounted on 10% agarose pads for imaging.

Recombinant proteins&nbsp;rMAb-611B1,&nbsp;rMAb-YL1/2, and rMAb-GT335 can be obtained from Kristen Verhey (kjverhey@umich) or can be produced based on the protein sequences in Blasius et al 2025 and Hotta et al 2026.&nbsp;

*Confocal Imaging*

Stained animals were mounted on 10% agarose pads for imaging at room temperature. Confocal imaging was performed using a Zeiss LSM 880 inverted microscope equipped with an Airyscan super-resolution module and ZEN Black software (Carl Zeiss Microscopy). Laser intensity was adjusted to avoid saturated pixels. Images were acquired using a 63×/1.4 oil Plan-Apochromat objective in SR mode. Image files were Airyscan processed and opened in ZEN Blue software or imported into Fiji (Schindelin et al., 2012) using the Bio-Formats Importer plugin (Linkert et al., 2010) for linear adjustment of contrast and creation of maximum intensity projections.

*CeNGEN Single-Cell RNA-seq Data Analysis*

Expression data for the α-tubulin acetyltransferase genes *atat-2* and *mec-17* were retrieved from the CeNGEN database (cengen.org; Hammarlund et al., 2018), which provides single-cell RNA sequencing (scRNA-seq) transcriptomes across *C.* *elegans* cell types, life stages, and sexes. Gene expression levels are reported as transcripts per million (TPM). Cell-type-specific expression profiles were examined across all available neuronal cell types; data from the highest-expressing neurons — CEP, OLQ, ALM, and PLM — are presented. Expression patterns were compared across larval and adult life stages in hermaphrodites and in adult males.

## Reagents

&nbsp;

**Table:** 

***C. elegans* Strain **	**Genotype**	**Available From**
PT3602	*cil-7 ( my61 [ cil-7 ::mNG])I; him-5 ( e1490 )V *	(Wang et al., 2021)
		
**Antibody**	**Clonality**	**Description**
rMAb-6-11B-1 (anti-α-tubulin K40 acetylation)	Recombinant monoclonal	Generated in (Blasius et al., 2025)
rMAb-YL1/2 (anti-tyrosinated α-tubulin)	Recombinant monoclonal	Generated in (Hotta et al., 2026)
rMAb-GT335 (anti-glutamylated tubulin)	Recombinant monoclonal	Generated in (Blasius et al., 2025)
